# Development of a diagnostic model for pre-washout screening of primary aldosteronism

**DOI:** 10.1007/s40618-024-02337-y

**Published:** 2024-03-27

**Authors:** Q. Wang, H. Dong, H.-W. LI, Z.-H. Zheng, Y.-Z. Liu, Y.-H. Hua, Y.-J. Xiong, H.-M. Zhang, L. Song, Y.-B. Zou, X.-J. Jiang

**Affiliations:** Department of Cardiology, State Key Laboratory of Cardiovascular Disease, Fuwai Hospital, National Center for Cardiovascular Diseases, Chinese Academy of Medical Sciences and Peking Union Medical College, No. 167 North Lishi Road, Beijing, 100037 Xicheng China

**Keywords:** Primary aldosteronism, Aldosterone, Renin, Screening, Aldosterone-to-renin ratio

## Abstract

**Purpose:**

Primary aldosteronism (PA) diagnosis is affected by antihypertensive drugs that are commonly taken by patients with suspected PA. In this study, we developed and validated a diagnostic model for screening PA without drug washout.

**Methods:**

We retrospectively analyzed 1095 patients diagnosed with PA or essential hypertension. Patients were randomly grouped into training and validation sets at a 7:3 ratio. Baseline characteristics, plasma aldosterone concentration (PAC), and direct renin concentration (DRC) before and after drug washout were separately recorded, and the aldosterone-to-renin ratio (ARR) was calculated.

**Results:**

PAC and ARR were higher and direct renin concentration was lower in patients with PA than in patients with essential hypertension. Furthermore, the differences in blood potassium and sodium concentrations and hypertension grades between the two groups were significant. Using the abbreviations potassium (P), ARR (A), PAC (P), sodium (S), and hypertension grade 3 (3), the model was named PAPS_3_. The PAPS_3_ model had a maximum score of 10, with the cutoff value assigned as 5.5; it showed high sensitivity and specificity for screening PA in patients who exhibit difficulty in tolerating drug washout.

**Conclusion:**

PA screening remains crucial, and standard guidelines should be followed for patients to tolerate washout. The PAPS_3_ model offers an alternative to minimize risks and enhance diagnostic efficiency in PA for those facing washout challenges. Despite its high accuracy, further validation of this model is warranted through large-scale clinical studies.

## Introduction

Primary aldosteronism (PA) is a major cause of secondary hypertension and accounts for 5–10% of all hypertension cases [1]. The clinically accepted indicator for PA screening is plasma aldosterone-to-renin ratio (ARR) [2], which is influenced by many factors, including age, medication usage, body position, time of blood collection, blood potassium levels, and salt intake [3–6]. Additionally, the ARR is affected by nearly all antihypertensive drugs commonly used in clinical practice; thus, there is a need to determine whether antihypertensive drugs affecting the renin–angiotensin–aldosterone system should be discontinued before screening [7, 8]. Certain drugs, such as diuretics, angiotensin-converting enzyme inhibitors (ACEIs), angiotensin receptor blockers (ARBs), β-adrenergic blockers, and calcium channel blockers (CCBs), should be discontinued before the measurement of ARR in patients with suspected PA [2]. However, discontinuing these drugs may lead to poor control of hypertension, interference with the treatment of other cardiovascular comorbidities, and difficulty tolerating drug washout due to postural hypotension and bradycardia in some patients. We investigated the effects of commonly used antihypertensive drugs on the plasma aldosterone concentration (PAC), direct renin concentration (DRC), and ARR of patients with PA and essential hypertension (EH). We also developed and validated a diagnostic model for pre-washout screening of PA using simple and easily available indicators. This model may mitigate the risk of blood pressure fluctuations caused by drug discontinuation or switching in some patients.

## Methods

### Study participants

A retrospective analysis of patients diagnosed with PA or EH at Fu Wai Hospital, Chinese Academy of Medical Sciences, was conducted between January 2016 and June 2022. The included patients were randomly divided at a 7:3 ratio, with 766 patients in the training set and 329 patients in the validation set. After establishing the model, external validation was performed using patients screened for hypertension etiology at our hospital from July 2022 to June 2023.

Inclusion criteria: patients with a final diagnosis of PA or EH who underwent upright PAC and DRC measurements before and after drug washout. All enrolled patients were from China, and there were no ethnic differences among them. Exclusion criteria:① patients aged > 65 years or < 18 years; ② those with secondary causes of hypertension, such as Cushing’s syndrome, pheochromocytoma, renal hypertension, renal artery stenosis, thyroid or parathyroid disease, or aortic disease; ③ pregnancy; ④ renal insufficiency; and ⑤ patients treated with mineralocorticoid receptor antagonists (MRA)[9] or estro-progestinic therapy before washout.

PA drug washout criteria [2]: attempt to correct blood potassium levels to normal range and maintain normal sodium intake; discontinuation of aldosterone receptor antagonists, diuretics, and licorice derivatives for at least 4 weeks before measurement; discontinuation of ACEIs, ARBs, CCBs, and β-adrenergic blockers for at least 2 weeks before measurement; and if blood pressure is poorly controlled, commencement of α-adrenergic blockers and non-dihydropyridine CCBs before measurement.

Conditions for blood collection for PA screening [2]: blood was collected midmorning after the patient had been up (sitting, standing, or walking) for at least 2 h and seated for 5–15 min. To minimize hemolysis, blood samples were maintained at room temperature (and not on ice) during delivery to the laboratory, with the plasma component rapidly frozen for storage after centrifugation. ARR was calculated from the PAC and DRC with units of ng/dL and mU/L, respectively, and ARR was reported in units (ng/dL)/(mU/L). Both PAC and DRC were measured using a chemiluminescent immunoassay.

Confirmation of PA diagnosis [2]: patients with post-washout ARR > 3.7 (ng/dL)/(mU/L) underwent the saline infusion test and captopril challenge test (CCT). After 4 h of continuous infusion, the saline infusion test was performed with 2000 mL of saline. PAC values < 5 ng/dL were considered negative, while values > 10 ng/dL were considered positive; values in between were considered inconclusive. The CCT was performed by administering 50 mg of captopril orally after sitting or standing for 1 h. PAC, DRC, and cortisol levels were measured before and 2 h after taking the drug. A PAC suppression of 30% was used as the cutoff value.

Hypertension grading criteria: according to the 2018 ESC/ESH Guidelines for the Management of Arterial Hypertension [10], grade 1 hypertension was defined as 140–159/90–99 mmHg (1 mmHg = 0.133 kPa), grade 2 hypertension as 160–179/100–109 mmHg, and grade 3 hypertension as ≥ 180/110 mmHg.

The study was observational and complied with the ethical principles of the Declaration of Helsinki for medical research involving human subjects. This study was approved by the Ethics Committee of Fuwai Hospital, Chinese Academy of Medical Sciences (No. 2016-802). All enrolled participants provided written informed consent.

### Study flow and group definitions

**Fig. 1:**
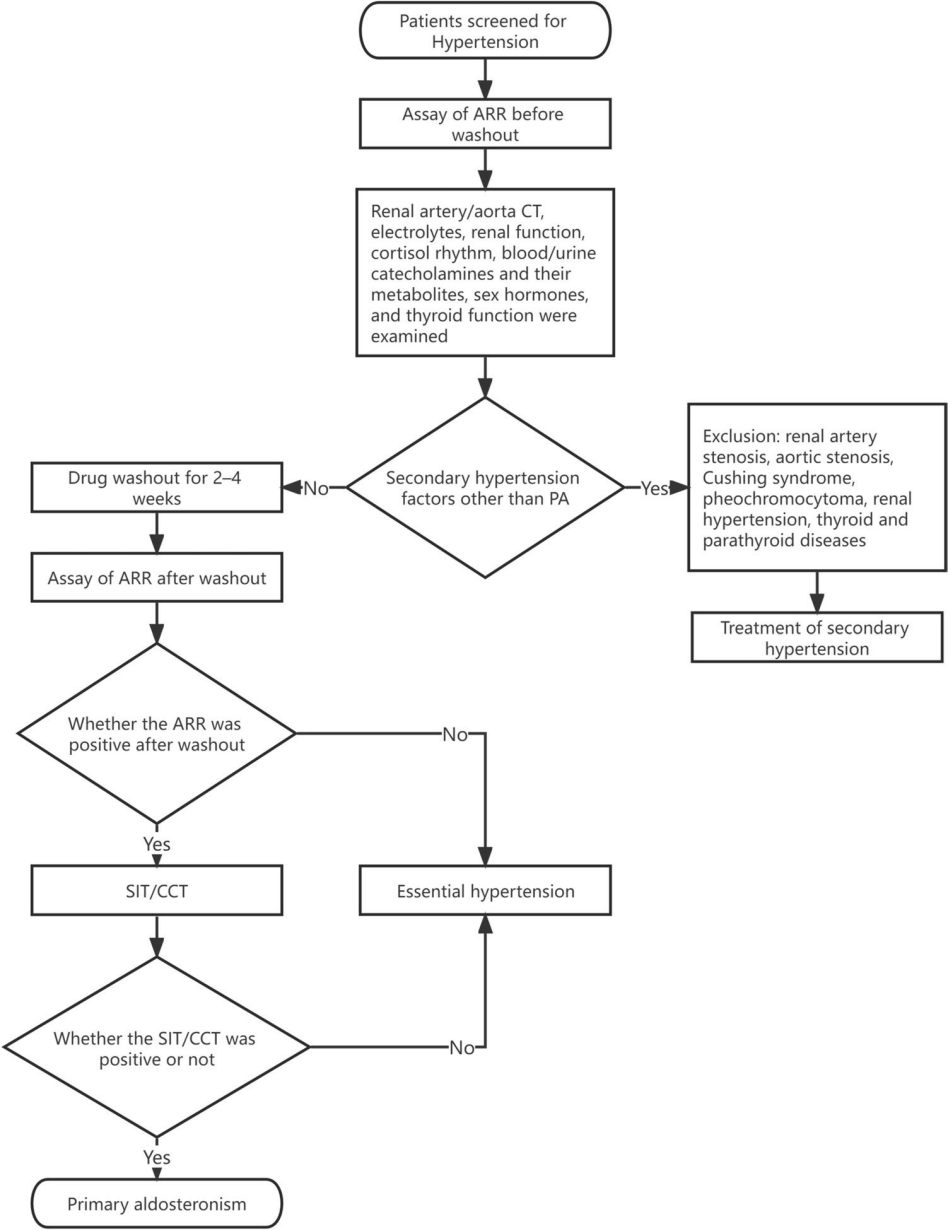
Flowchart of screening patients with hypertension. *ARR* aldosterone/renin ratio, *CT* computed tomography, *PA* primary aldosteronism, *SIT* saline infusion test, *CCT* captopril challenge test

**Fig. 2:**
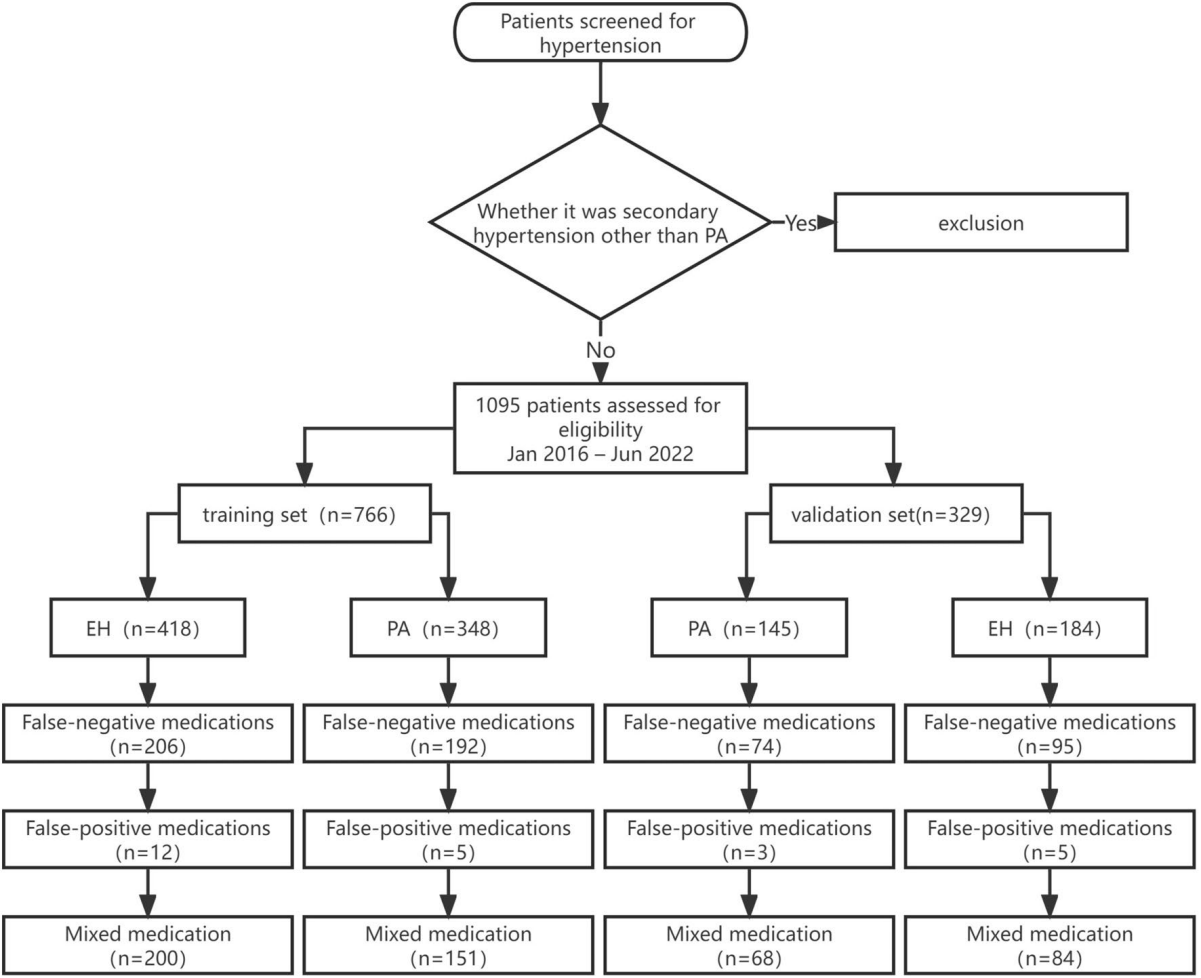
Flowchart of study methods. *PA* primary aldosteronism, *EH* essential hypertension, *ARR* aldosterone/renin ratio

A flowchart of the hypertension screening process and specific study groups are shown in Figs. 1 and 2, respectively. False-negative medications included ACEIs, ARBs, CCBs, and diuretics; false-positive medications included β-adrenergic blockers. Mixed medication was defined as the simultaneous use of false-negative and –positive medications. Blood potassium concentration was the lowest value recorded during the patient’s visits. The blood sodium concentration was measured simultaneously as the lowest blood potassium value.

### Statistical analysis

SPSS 26.0 software (IBM Corp., Armonk, NY) was used for statistical analysis. Measurement data were expressed as the mean ± standard deviation or median with interquartile range, and comparisons were conducted using Student’s t-tests or Mann–Whitney U tests. Count data were expressed as frequencies and percentages, and the χ^2^ test was used to compare groups. The training and validation sets were randomly assigned at a 7:3 ratio. In the training set, the optimal cutoff value was determined using the receiver operating characteristic (ROC) curve to convert the alternative predictors into binary variables; binary logistic regression analysis was used to determine the predictors for the pre-washout screening of PA. Each predictor was assigned a value by rounding β to the nearest whole number and then setting up a threshold, thereby constructing a model for PA screening. The Hosmer–Lemeshow test was used to assess the model’s goodness-of-fit, ROC curve analysis was used to assess the screening efficacy of the model, and internal bootstrapping validation was used to assess the stability of the model. Two-sided tests were used, and differences with *p* < 0.05 were considered statistically significant.

## Results

### Baseline characteristics of patients with PA and EH

A total of 1095 patients who underwent testing between January 2016 and June 2022 were enrolled in the study. The mean patient age was 53.0 ± 12.0 years and 57.0% were male. The patients had an average hypertension grade of 2.5 ± 0.7. The specific classes of antihypertensive drugs included dihydropyridine CCBs in 58.7% of patients, ACEIs or ARBs in 47.5%, β-adrenergic blockers in 24.1%, diuretics in 10.1%, non-dihydropyridine CCBs in 6.8%, and α-adrenergic blockers in 6.1%. The baseline characteristics of patients with PA and EH are listed in Table 1.

**Table 1:** Baseline characteristics and comparison of patients with primary aldosteronism and essential hypertension

Variable	Total (n = 1095)	PA (n = 493)	EH (n = 602)	*p* value
Age ($\overline{X}$ ± SD, years)	53.0 ± 12.0	52.5 ± 10.6	53.5 ± 12.9	< 0.001
Duration of disease ($\overline{X}$ ± SD, years)	10.5 ± 9.1	10.5 ± 9.0	12.0 ± 13.4	0.168
Male sex [cases, (%)]	624 (57.0)	267 (54.2)	357 (59.3)	0.098
BMI ($\overline{X}$ ± SD, kg/m^2^)	26.48 ± 4.05	26.76 ± 4.02	26.25 ± 4.06	0.889
Hypertension grade ($\overline{X}$ ± SD, grade)	2.5 ± 0.7	2.7 ± 0.5	2.4 ± 0.7	< 0.001
Refractory hypertension [cases, (%)]	94 (8.6)	87 (17.6)	7 (1.2)	< 0.001
Number of antihypertensive drugs ($\overline{X}$ ± SD, number)	1.5 ± 1.1	2.0 ± 1.1	1.1 ± 1.0	0.046
3 drugs [cases, (%)]	181 (16.5)	136 (27.6)	45 (7.5)	< 0.001
≥ 4 drugs [cases, (%)]	40 (3.6)	36 (7.3)	4 (0.7)	< 0.001
Blood potassium ($\overline{X}$ ± SD, mmol/L)	3.69 ± 0.50	3.40 ± 0.50	3.93 ± 0.34	< 0.001
Blood sodium ($\overline{X}$ ± SD, mmol/L)	142.29 ± 2.73	143.76 ± 2.79	141.08 ± 1.99	0.007
Blood creatinine ($\overline{X}$ ± SD, μmol/L)	82.60 ± 23.98	83.66 ± 21.96	81.74 ± 25.51	0.388

*PA* primary aldosteronism, *EH* essential hypertension, *SD* standard deviation, *BMI* body mass index

A *p* value < 0.05 was considered statistically significant

Among the 1095 patients included, 493 had PA (45.0%) and 602 had EH (55.0%). Comparison between the patients with PA and EH indicated that the differences in the proportion of male patients (*p* = 0.098), duration of disease (*p* = 0.168), body mass index (BMI) (*p* = 0.889), and blood creatinine (*p* = 0.388) between groups were not significant. By contrast, differences in age (52.5 ± 10.6 vs. 53.5 ± 12.9 years; *p* < 0.001), hypertension grade (2.7 ± 0.5 vs. 2.4 ± 0.7; *p* < 0.001), proportion of patients with refractory hypertension (17.6 vs. 1.2%; *p* < 0.001), number of classes of antihypertensive drugs taken (2.0 ± 1.1 vs. 1.1 ± 1.0 classes; *p* = 0.046), blood sodium concentration (143.76 ± 2.79 vs. 141.08 ± 1.99 mmol/L; *p* = 0.007), and blood potassium concentration (3.40 ± 0.50 vs. 3.93 ± 0.34 mmol/L; *p* < 0.001) were significant. The comparison between patients with PA and EH is shown in Table 1.

### Comparison of PAC, DRC, and ARR values under different conditions

**Fig. 3:**
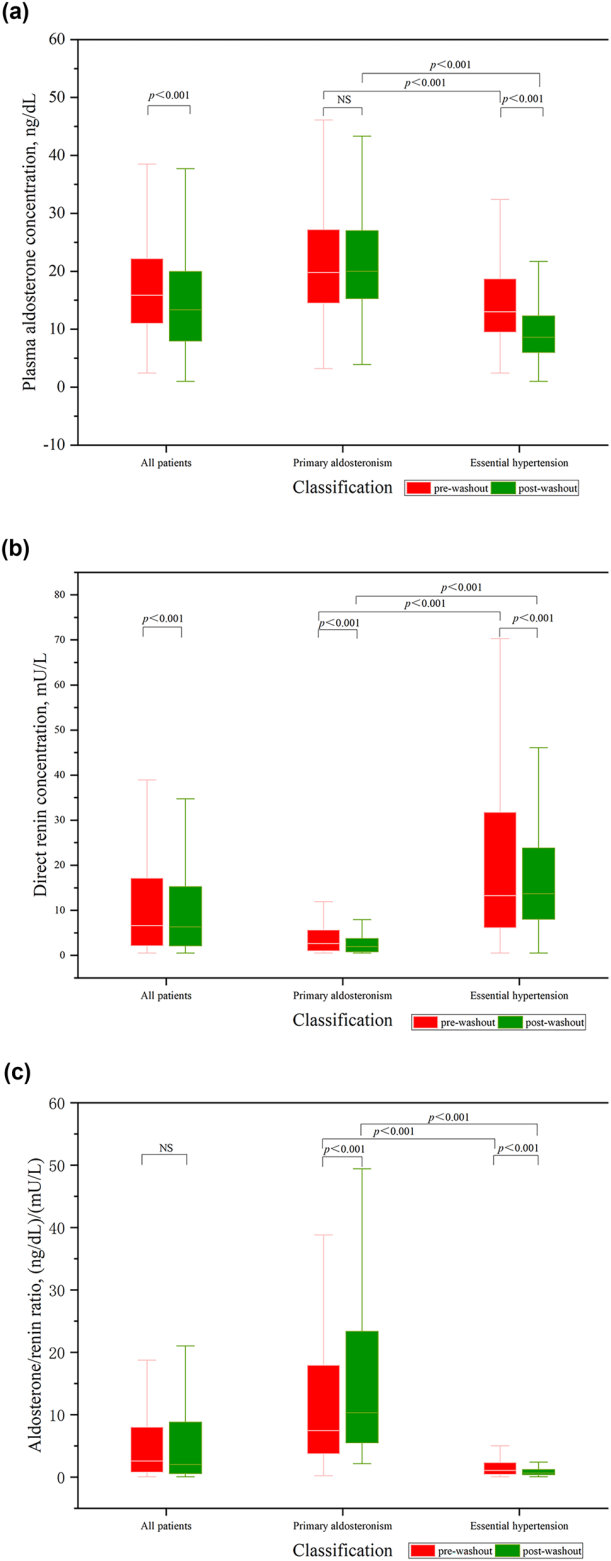
Comparison of the plasma aldosterone concentration (PAC), direct renin concentration (DRC), and aldosterone/renin ratio (ARR) values between different groups. **a** In all patients, the differences in the PAC [15.85 (11.00, 22.20) vs. 13.35 (7.92, 19.98) ng/dL; *p* < 0.001] values before and after washout were significant. In patients with PA, comparisons of the PAC (*p* = 0.060) values indicated no significant difference before and after washout. The pre-washout PAC [19.80 (14.48, 27.20) vs. 13.00 (9.48, 18.70) ng/dL; *p* < 0.001] values were significantly higher in patients with PA than in patients with EH. The post-washout PAC [20.00 (15.30, 27.02) vs. 8.60 (6.00, 12.30) ng/dL; *p* < 0.001) values were significantly higher in patients with PA than in patients with EH. **b** The differences in the DRC [(6.58 (2.10, 17.13) vs. 6.30 (2.10, 15.20) mU/L; *p* < 0.001] values before and after washout were significant in all patients. In patients with PA, comparisons of the DRC [2.60 (1.00, 5.65) vs. 1.90 (0.80, 3.70) mU/L; *p* < 0.001], values indicated a significant difference before and after washout. The pre-washout DRC [2.60 (1.00, 5.65) vs. 13.25 (6.09, 31.80) mU/L; *p* < 0.001] levels were significantly lower in patients with PA than in patients with EH. The post-washout DRC [1.90 (0.80, 3.70) vs. 13.65 (7.98, 23.80) mU/L; *p* < 0.001] values were lower in patients with PA than in patients with EH. **c** The difference in ARR (*p* = 0.552) was not significant in all patients. In patients with PA, comparisons of the ARR [7.43 (3.71, 17.96) vs. 10.29 (5.45, 23.40) [ng/dL]/[mU/L]; *p* < 0.001] values indicated a significant difference before and after washout. The pre-washout ARR [7.43 (3.71, 17.96) vs. 1.05 (0.41, 2.33) [ng/dL]/[mU/L]; *p* < 0.001] values were significantly higher in patients with PA than in patients with EH. The post-washout ARR [10.29 (5.45, 23.40) vs. 0.62 (0.37, 1.21) [ng/dL]/[mU/L], *p* < 0.001] values were significantly higher in patients with PA than in patients with EH

Among 1095 patients, the positive concordance rate of ARR before and after washout was 79.7%, the negative concordance rate was 81.4%, and the diagnostic concordance rate of ARR was 80.6%. The misdiagnosis rate was 15.4%, and the missed diagnosis rate was 23.5%. In all patients, the differences in the PAC [15.85 (11.00, 22.20) vs. 13.35 (7.92, 19.98) ng/dL; *p* < 0.001] and DRC [(6.58 (2.10, 17.13) vs. 6.30 (2.10, 15.20) mU/L; *p* < 0.001] values before and after washout were significant, whereas the difference in ARR (*p* = 0.552) was not significant. In patients with PA, comparisons of the PAC (*p* = 0.060), DRC [2.60 (1.00, 5.65) vs. 1.90 (0.80, 3.70) mU/L; *p* < 0.001], and ARR [7.43 (3.71, 17.96) vs. 10.29 (5.45, 23.40) [ng/dL]/[mU/L]; *p* < 0.001] values indicated a significant effect on DRC and ARR before and after washout. The pre-washout PAC [19.80 (14.48, 27.20)vs. 13.00 (9.48, 18.70) ng/dL; *p* < 0.001] and ARR [7.43 (3.71, 17.96) vs. 1.05 (0.41, 2.33) [ng/dL]/[mU/L]; *p* < 0.001] values were significantly higher, while the pre-washout DRC [2.60 (1.00, 5.65) vs. 13.25 (6.09, 31.80) mU/L; *p* < 0.001] levels were significantly lower in patients with PA than in patients with EH. Similarly, the post-washout PAC [20.00 (15.30, 27.02) vs. 8.60 (6.00, 12.30) ng/dL; *p* < 0.001] and ARR [10.29 (5.45, 23.40) vs. 0.62 (0.37, 1.21) [ng/dL]/[mU/L], *p* < 0.001] values were significantly higher, while the DRC [1.90 (0.80, 3.70) vs. 13.65 (7.98, 23.80) mU/L; *p* < 0.001] values were lower in patients with PA than in patients with EH. Figure 3 shows the comparisons of PAC, DRC, and ARR under different groups.

Among the 567 patients who were only administered false-negative drugs, there were 300 cases of single-drug use, 199 cases of dual-drug use, and 68 cases of triple- or multi-drug use. Among the 300 patients with single-drug use, 84 used ACEIs/ARBs, 210 used CCBs, and 6 used diuretics. Using the sample data, we compared the changes in PAC, DRC, and ARR before and after washout in two groups with a relatively large sample size. These groups were patients using ACEIs/ARBs alone and patients using CCBs alone. For patients using ACEIs/ARBs, comparing the ARR values before and after washout, we found that this class of drugs significantly increased pre-washout DRC (*p* < 0.001) and expressively decreased pre-washout PAC (*p* = 0.038) and ARR (*p* = 0.014). At the same time, we observed that patients using ACEIs/ARBs, who tested positive for ARR before washout, continued to test positive for ARR after washout, with a false positive rate of 0%. For patients using CCBs alone, although there was a trend of decreasing PAC (*p* = 0.061), and increasing DRC (*p* = 0.303), resulting in decreased in ARR before washout (*p* = 0.248), none of these differences reached statistical significance (*p* > 0.05). Table 2 shows the comparison of PAC, DRC, and ARR values under different false-negative drugs.

**Table 2:** Comparison of the plasma aldosterone concentration, direct renin concentration, and aldosterone/renin ratio values in all patients receiving different false negative drugs

Classification	Variable	Pre-washout	Post-washout	*p* value	False-positive (%)	False-negative (%)
ACEIs/ARBs (n = 84)						
PAC [M (P25, P75), ng/dL]		11.20 (7.90, 18.00)	14.10 (9.67, 19.80)	0.038	0	18.18
DRC [M (P25, P75), mU/L]		24.59 (6.07, 65.42)	11.70 (3.25, 23.55)	< 0.001		
ARR [M (P25, P75), (ng/dL)/(mU/L)]		0.44 (0.17, 2.65)	1.05 (0.45, 2.89)	0.014		
CCBs (n = 210)						
PAC [M (P25, P75), ng/dL]		11.85 (6.98, 18.68)	16.50 (10.98, 22.13)	0.061	14.39	33.33
DRC [M (P25, P75), mU/L]		9.00 (3.20, 22.30)	7.95 (2.70, 15.97)	0.303		
ARR [M (P25, P75), (ng/dL)/(mU/L)]		1.15 (0.42, 6.22)	2.20 (1.04, 6.04)	0.248		

*ACEIs* angiotensin-converting enzyme inhibitors, *ARBs* angiotensin receptor blockers, *CCBs* calcium channel blockers, *PAC* plasma aldosterone concentration, *DRC* direct renin concentration, *ARR* aldosterone/renin ratio, *M* median, *P25* the first quartile, *P75* the third quartile

False-Positive: The number of patients with a positive pre-wash ARR but negative post-wash ARR was divided by the total number of patients with negative post-wash ARR, multiplied by 100%

False-Negative: The number of patients with a negative pre-wash ARR but positive post-wash ARR was divided by the total number of patients a with positive post-wash ARR, multiplied by 100%

A *p* value < 0.05 was considered statistically significant

Subgroup analysis of patients in the PA group was performed based on the classes of drugs used before washout. The patients in the false-negative drug group had significantly higher DRC values pre-washout than post-washout (*p* < 0.001), whereas the ARR pre-washout was significantly lower than post-elution (*p* < 0.001); the difference between the pre- and post-washout PAC was not significant (*p* = 0.144). The patients in the false-positive drug group had no significant differences in pre- and post-washout PAC (*p* = 0.556), DRC (*p* = 0.151), or ARR (*p* = 0.932) values. The patients in the mixed drug group had higher pre-washout than post-washout DRC values (*p* = 0.002), but the differences in pre- versus post-washout PAC (*p* = 0.362) and ARR (*p* = 0.054) values were not significantly different. Table 3 presents a comparison of PAC, DRC, and ARR values in patients with PA under different drug treatments.

**Table 3:** Comparison of the plasma aldosterone concentration, direct renin concentration, and aldosterone/renin ratio values in patients with primary aldosteronism receiving different drugs

Classification	Variable	Pre-washout	Post-washout	*p* value
False-negative drug group (n = 266)				
PAC [M (P25, P75), ng/dL]		20.10 (14.70, 28.00)	20.50(15.50, 28.00)	0.144
DRC [M (P25, P75), mU/L]		2.80 (1.10, 6.85)	1.90 (0.85, 3.60)	< 0.001
ARR [M (P25, P75), (ng/dL)/(mU/L)]		7.30 (3.26, 16.95)	10.30 (5.75, 24.05)	< 0.001
False-positive drug group (n = 8)				
PAC [M (P25, P75), ng/dL]		21.90 (16.10, 30.20)	24.9 (14.70, 34.40)	0.556
DRC [M (P25, P75), mU/L]		1.80 (0.50, 4.70)	2.90 (0.50, 7.00)	0.151
ARR [M (P25, P75), (ng/dL)/(mU/L)]		10.88 (4.89, 32.20)	12.48 (3.77, 17.60)	0.932
Mixed drug group (n = 219)				
PAC [M (P25, P75), ng/dL]		19.15 (14.08, 26.63)	19.70 (15.08, 26.20)	0.362
DRC [M (P25, P75), mU/L]		2.40 (0.90, 4.63)	2.00 (0.80, 3.80)	0.002
ARR [M (P25, P75), (ng/dL)/(mU/L)]		7.49 (4.21, 21.44)	10.16 (5.33, 22.60)	0.054

*PAC* plasma aldosterone concentration, *DRC* direct renin concentration, *ARR* aldosterone/renin ratio, *M* median, *P25* the first quartile, *P75* the third quartile

A *p* value < 0.05 was considered statistically significant

### Construction of a pre-washout PA screening model

**Fig. 4:**
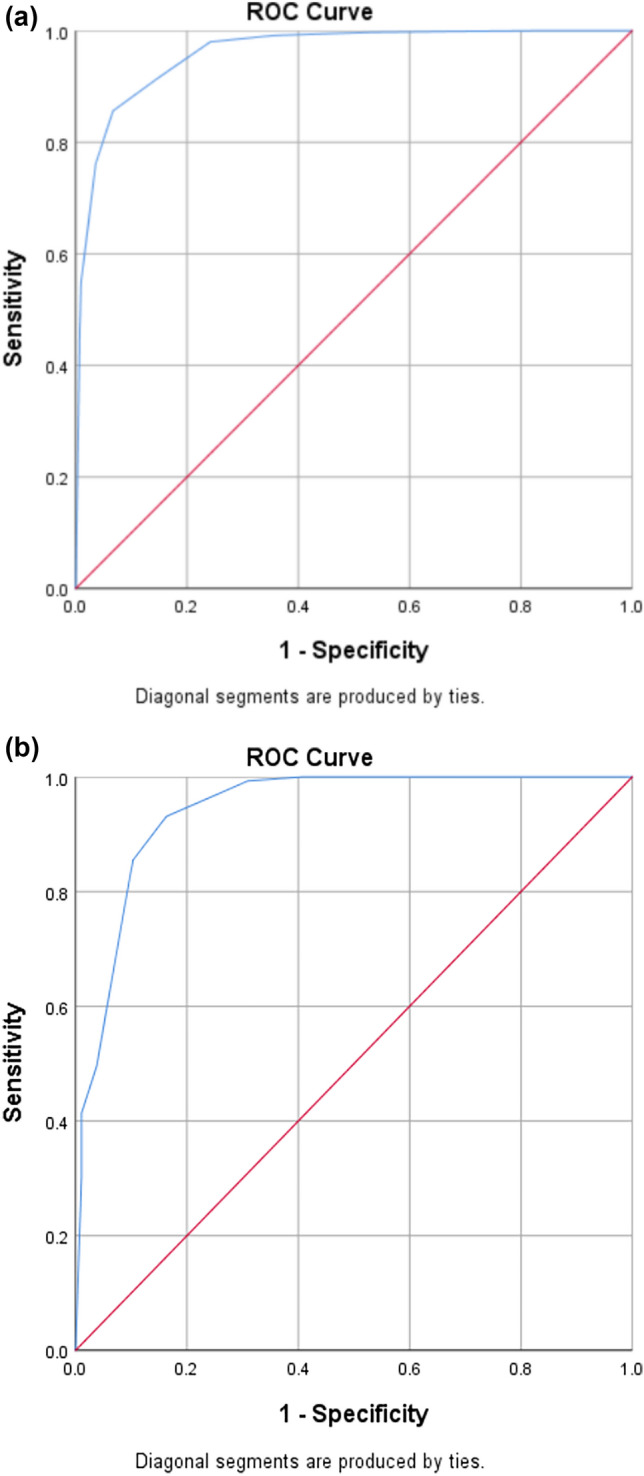
Receiver operating characteristic (ROC) curves of the PAPS_3_ model. **a** ROC curve of the patients in the training set. AUC: 0.961 (95% CI 0.949–0.973). **b** ROC curve of the patients in the validation set. AUC: 0.943 (95% CI 0.919–0.966)

A pre-washout PA screening model was prepared for patients who could not tolerate drug washout. The 1095 patients were included and randomly divided at a 7:3 ratio into training (766 patients) and validation set (329 patients); the difference in the proportion of patients with PA between the training and validation sets (45.4% vs. 44.1%, respectively; *p* = 0.691) was not significant (Fig. 2). A comparison of the patients with PA and EH revealed significant differences in hypertension grade, blood potassium, blood sodium, and pre-washout PAC, DRC, and ARR values; these were included as alternative predictors and converted to binary variables by determining the cutoff values using ROC curve analysis. Finally, pre-washout ARR > 2.60 (ng/dL)/(mU/L), PAC > 14.00 ng/dL, DRC < 6.20 mU/L, blood potassium < 3.50 mmol/L, blood sodium > 142.00 mmol/L, and hypertension grade 3 were included in the binary logistic regression analysis (Table 4). Based on their β values, blood potassium < 3.50 mmol/L and ARR > 2.60 (ng/dL)/(mU/L) were assigned three points each, blood sodium > 142.00 mmol/L was assigned two points, and PAC > 14.00 ng/dL and hypertension grade 3 were assigned one point each. Using the abbreviations potassium (P), ARR (A), PAC (P), sodium (S), and hypertension grade 3 (3), the model was named PAPS_3_ and had a maximum score of 10. The cutoff value of the PAPS_3_ model was determined to be 5.5 through ROC curve analysis at the maximum Youden index. The model had a sensitivity of 85.6% and a specificity of 92.3% for screening PA. The area under the ROC curve of the model was 0.961 (95% confidence interval [CI] 0.949–0.973) (Fig. 4).

**Table 4:** Binary logistic regression analysis of primary aldosteronism predictors

Variable	*β*	Wald statistic	*p* value	OR (95% CI)
PAC > 14.00 ng/dL	1.178 (1 point)	15.824	< 0.001	3.248 (1.818–5.803)
DRC > 6.20 mU/L	0.754	2.330	0.127	2.127 (0.807–5.603)
ARR > 2.60 (ng/dL)/(mU/L)	2.697 (3 points)	29.449	< 0.001	14.829 (5.599–39.273)
Blood potassium < 3.50 mmol/L	2.569 (3 points)	73.646	< 0.001	13.048 (7.257–23.459)
Hypertension grade 3	1.241 (1 point)	18.441	< 0.001	3.459 (1.963–6.095)
Blood sodium > 142.00 mmol/L	2.216 (2 points)	63.708	< 0.001	9.167 (5.320–15.795)

*PAC* plasma aldosterone concentration, *DRC* direct renin concentration, *ARR* aldosterone/renin ratio, *OR* odds ratio, *CI* confidence interval

A *p* value < 0.05 was considered statistically significant

The screening model was calibrated using the Hosmer–Lemeshow test, yielding a test statistic of 6.490 (df = 8, *p* = 0.593). Internal bootstrapping validation was then performed to assess the stability of the model, and the predictors obtained from internal bootstrapping validation (1000 times) were consistent with the model derived from the original data, with an area under the ROC curve of 0.961 (95% CI 0.949–0.973).

Evaluation of the predictive power of the special population in the training set revealed that the area under the ROC curve for patients taking false-negative drugs was 0.962 (95% CI 0.945–0.979), and the model had a sensitivity of 84.9% and a specificity of 93.7% for the screening of PA. Additionally, the area under the ROC curve for patients taking mixed drugs (false-negative + false-positive) was 0.963 (95% CI 0.946–0.980), and the model had a sensitivity of 86.8% and a specificity of 93.0% for the screening of PA.

### Validation of the pre-washout PA screening model

In the validation set, the area under the ROC curve of the screening model was 0.943 (95% CI 0.919–0.966) (Fig. 4). The cutoff value of the model was 5.5, and the model had a sensitivity of 85.5% and a specificity of 89.7% for positive screening of PA. The model demonstrated an accuracy of 82.8%, a positive predictive value of 86.7%, a negative predictive value of 88.7%, a misdiagnosis rate of 10.3%, and a missed diagnosis rate of 14.5%. Evaluation of the predictive power of the special population in the validation set revealed that the area under the ROC curve for patients taking false-negative drugs was 0.951 (95% CI 0.920–0.981), and the model had a sensitivity of 87.8% and a specificity of 89.5% for the screening of PA. Additionally, the area under the ROC curve for patients taking mixed drugs (false-negative + false-positive) was 0.930 (95% CI 0.891–0.970), and the model had a sensitivity of 82.4% and a specificity of 89.3% for the screening of PA. After establishing the PAPS_3_ model, we conducted validation over one year by using the model on patients who visited our hospital for hypertension screening from July 2022 to June 2023. After excluding other secondary hypertension factors, a total of 191 patients were selected. Results from the final validation of the PAPS_3_ model showed that the sensitivity was 89.1%, specificity was 88.9%, misdiagnosis rate was 11.1%, and missed diagnosis rate was 10.9%.

## Discussion

PA is a common but severely underdiagnosed form of secondary hypertension [11, 12]. Elevated aldosterone levels are strongly associated with cardiovascular events and metabolic syndrome, and patients with PA are often prone to severe organ damage [13, 14]. Therefore, early detection and treatment of PA are necessary to minimize cardiovascular complications. Screening for PA is usually a multi-step process that uses ARR measurement as the initial screening condition [15]. In 2008, the European Society of Endocrinology published guidelines for the diagnosis and treatment of PA [16], recommending the use of ARR as a screening indicator; international and domestic guidelines and expert consensuses currently also recommend ARR as a screening index [2]. Additionally, drugs affecting the renin–angiotensin–aldosterone system should be discontinued before ARR measurement to improve the accuracy of PA screening [17]. In patients with hypertension, non-dihydropyridine CCBs and α-adrenergic blockers should be administered to control blood pressure. In this study, the missed diagnosis rate due to pre-washout ARR was nearly 25.0%. This highlights the importance of performing a repeat analysis of ARR after medication washout. However, these recommendations are not easy to implement universally, especially in patients with severe hypertension, where the hypotensive effects of drug washout are limited and patients cannot tolerate an entire 2–4 week washout. Additionally, some patients cannot tolerate non-dihydropyridine CCBs or α-adrenergic blockers owing to bradycardia or severe postural hypotension, making drug washout for PA screening difficult. Therefore, a complete washout before PA screening is difficult. Herein, 55.9% of patients (612/1095) were treated with two or more antihypertensive drugs to control blood pressure, further complicating drug washout. Consequently, simplifying the process of PA screening is an urgent clinical need to ensure patient safety and reduce blood pressure fluctuations while also improving the sensitivity and specificity of diagnosis. Thus, we included a retrospective analysis of ARR in 1095 patients who underwent hypertension screening and measured their PAC and DRC values before and after washout. The ARR values were analyzed to construct a screening model suitable for pre-washout PA screening.

Many clinical factors affect the ARR and unifying them is difficult. In this study, patients aged > 65 years or < 18 years and those with renal insufficiency were excluded, as were patients with factors such as renal hypertension, renal artery stenosis, and pregnancy, which interfere with ARR. Patients were instructed to maintain normal sodium intake throughout the screening process, and efforts were made to correct blood potassium levels to within the normal range; this was done to minimize the impact of blood potassium and sodium intake on ARR and prevent the impact of factors unrelated to medication on ARR.

Among the included patients, 45% had PA and 55% had EH. There was no significant difference in the sex ratio, duration of disease, BMI, or blood creatinine level between the two groups. However, the two groups had significant differences in age, hypertension grade, proportion of patients with refractory hypertension, number of antihypertensive drugs used, and blood sodium and potassium concentrations. Patients with PA were younger, had higher blood pressure, were poorly controlled with medication, and had lower blood potassium and higher blood sodium levels than patients with EH. Although recent studies showed that PA could present as hypertension with normal blood potassium, accounting for approximately 39–50% of all patients with PA [18, 19], hypokalemia cannot be used as a diagnostic criterion for PA [20] nor can normal blood potassium be used as an exclusion criterion. However, some studies have shown that the prevalence of PA is as high as 88.5% in patients with spontaneous hypokalemia and blood potassium concentrations < 2.5 mmol/L [21]. The prevalence of hypokalemia among Chinese patients with PA has been reported to be 74.8% [22]. Herein, the mean blood potassium concentration of the patients with PA was 3.40 ± 0.50 mmol/L, which was lower than normal, and the proportion of those with hypokalemia was 69.6%. This indicates that over half of the patients with PA had hypokalemia as the primary manifestation in addition to elevated blood pressure, suggesting that blood potassium levels can be considered adjunct markers in pre-washout screening. Hypertensive patients with a history of hypokalemia are more likely to be of clinical concern, and the retrospective design of the study (including only patients screened for PA based on clinical suspicion) may carry an element of selection bias, leading to an overestimation of the performance of the diagnostic model for subsequent screening [23].

Before washout, patients with PA still exhibited higher PAC, lower DRC, and higher ARR values than patients with EH (Fig. 3), indicating that these clinical manifestations of PA remained prominent despite medication acting as a confounding factor. Moreover, these indicators can be included as alternative predictors in the development of the screening model. To validate the effects of medication on the PAC, DRC, and ARR values during PA screening, we compared these values in patients with PA before and after washout. We found that the PAC levels did not change significantly before and after washout; however, the DRC levels were higher before washout, leading to decreased ARR before washout (Fig. 3). This finding indicates that drug interference primarily affects the DRC and produces a false-negative ARR result. Next, patients with PA were divided into false-negative, false-positive, and mixed drug groups based on the classes of drugs they used before washout. No significant differences between the pre-and post-washout PAC values were observed in all three groups (Table 3). In both the false-negative and mixed drug groups, DRC values decreased after washout (*p* < 0.05); however, the only significant difference was the elevation in post-washout ARR in the false-negative drug group (Table 3). Considering that the pre-washout drugs taken by the patients here were ACEIs/ARBs and CCBs and 10% of the patients were treated with diuretics, the drug-associated interfering factors were primarily due to false-negative drugs. Mixed drugs were also common, whereas using false-positive drugs alone was rare; this resulted in the drugs having both increasing and decreasing effects on the PAC values of patients with PA, leading to no significant differences. The primary effect of medication was a significant increase in the DRC value. The ARR was affected by these changes in PAC and DRC, resulting in either increased or decreased values, and there was a significant difference in the false-negative drug group. Additionally, although the pre-washout DRC values were elevated in the false-negative drug group, the absolute value of DRC did not increase significantly, possibly because DRC is affected by PA itself in addition to the effects of medication, and the overlap of these two factors attenuated the effects of medication. The sample size of the false-positive drug group was small, and there were no significant differences in the PAC, DRC, or ARR values before and after washout (Table 3). This result suggests that the sample size should be expanded in future studies to confirm the present results. Additionally, we conducted an in-depth analysis of false-negative drugs and made several inferences: ① ACEIs/ARBs mainly affect DRC, causing a decrease in ARR, with no significant impact on PAC. If ACEIs/ARBs are used and the pre-washout ARR is positive, it is speculated that it will remain positive after washout; ② CCBs have no significant impact on PAC and DRC, and there is no significant change in ARR before and after washout. For patients who find it challenging to complete washout, considering the use of CCB drugs may be an option; ③ Subgroup analysis for the above-mentioned false-negative drugs suggests that they have no significant impact on PAC. Therefore, for patients using these drugs, regardless of whether their ARR results are positive or not, attention should be paid to abnormal elevation of PAC to reduce missed diagnoses; ④ In patients treated with only ACEIs/ARBs here, the mechanisms of action of the drugs on ARR and PAC were the same as those in the CCT, in which PAC decreased and DRC increased. Thus, using this class of drugs could potentially lead to an increased probability of false-negative ARR values [24, 25]. A diagnosis of PA can be considered in the case of positive ARR and PAC results upon treatment with ACEIs/ARBs alone.

There were differences between the patients with PA and EH in terms of the classes of medication used. Because this was a retrospective study, the classes, duration, and doses of medication were not standardized during consultation and treatment; hence, achieving uniformity and standardization during follow-up consultation and treatment was difficult. The pre-washout treatment status was determined without uniform standardized drug treatment; therefore, we concluded that these indicators were subjectively influenced and unsuitable for inclusion as alternative predictors. Additionally, although there was a significant difference in the age between patients with PA and EH, the absolute age difference between the two groups was not significant nor was age considered an alternative predictor, possibly owing to the sample size. Taking these factors together, six indicators were selected: blood potassium; blood sodium; hypertension grade; pre-washout PAC, DRC, and ARR, all exhibiting significant differences between PA and EH. These values were clinically easy to obtain and yielded relatively stable test results.

The determination of cutoff values for these predictors using ROC curve analysis, subsequent conversion to binary variables, and logistic regression analysis were used to construct the PAPS_3_ screening model. The factors included in this model are also consistent with the predictors for PA screening currently used in most institutions [4, 26, 27]. Although most studies have not set an additional threshold for PAC when using ARR for PA screening, some have set PAC thresholds [4, 8, 26, 28], including PAC ≥ 9 ng/dL [26] and PAC > 16 ng/dL [28]. The PAPS_3_ model had a maximum score of 10, a cutoff value of 5.5, a sensitivity of 85.6%, and a specificity of 92.3% for screening PA. Calibration of the PAPS_3_ model was further performed using the Hosmer–Lemeshow test, which indicated a good fit with the original data and good predictive power. The stability of the PAPS_3_ model was further evaluated using internal bootstrapping validation, and the results confirmed that the model was stable.

The PAPS_3_ model showed high sensitivity, specificity, and accuracy, with an area under the curve > 0.9. There are many possible reasons for this. First, of the five indicators selected for the construction of the model, three (hypertension grade, blood potassium, and blood sodium) were not restricted to pre- or post-washout or affected by drugs before or after washout. Additionally, there was no significant difference in the PAC of patients with PA before and after washout. Subgroup analysis also indicated no significant difference in the PAC values between the drug treatment groups. This suggests that pre- and post-washout PAC was unaffected by large fluctuations caused by drug interference. As an indicator in the primary screening of PA, ARR plays a key role in the diagnostic process. Using ARR > 2.60 (ng/dL)/(mU/L) as the cutoff value, the present study found a consistency of 80.8% between pre- and post-washout ARR, indicating a high degree of overlap. Therefore, based on these factors, the PAPS_3_ model had high accuracy.

Despite the model’s accuracy, we also considered potential drawbacks during its development. First, the model was based on a retrospective analysis; all included patients had undergone drug washout. No patients who had difficulty tolerating drug washout were included, thereby introducing selection bias; however, there is currently no clinical solution to this problem. Additionally, patients with concomitant hypertension and hypokalemia, as well as those with pre-washout abnormalities in PAC and ARR, are more likely to draw the attention of clinicians and undergo proper PA screening; this bias can significantly inflate the measures on diagnostic tests [23]. Furthermore, a growing body of evidence shows that PA is present in some cases of mild hypertension and even in populations with normal blood pressure. These patients often do not receive further PA screening and are more likely to undergo follow-up only in primary care settings such as community clinics [29–31], causing some patients with PA to be missed. Additionally, there is great variability between study designs that is difficult to reconcile; variables include the population of interest, ARR threshold value, methods of renin and aldosterone measurement, and type and diagnostic thresholds of the confirmatory tests used, all of which introduce variations in the sensitivities and specificities of PA screening reported in different studies. Finally, a “gray area” in diagnosing PA makes a definitive diagnosis of EH or PA difficult.

In the past, researchers constructed similar models for the classification and diagnosis of PA. The Küpers score [32] is a typical predictive model for the staging of PA that includes typical adenoma imaging with thresholds of blood potassium < 3.5 mmol/L and glomerular filtration rate > 100 mL/(min. 1.73 m^2^). At Küpers score ≥ 5, the sensitivity and specificity for diagnosing unilateral PA are 53% and 100%, respectively. However, a Chinese study found that the Küpers score had low sensitivity and specificity for the Chinese population (62% and 53%, respectively), which did not apply to the elderly population. After modifying the model, it was found that urinary aldosterone levels, history of hypokalemia, and typical unilateral adenoma diameter > 1 cm had a diagnostic specificity of 90.5%. In China, He et al*.* used imaging histology techniques and clinical characteristics to establish a nomogram model that includes adrenal computed tomography imaging histology score, age, sex, blood potassium, and ARR to predict aldosterone-producing adenoma [33]. Although the PAPS_3_ model cannot distinguish the specific stage of PA, its advantages include the use of simple and easily obtainable predictors, convenience for clinical application, and high sensitivity and specificity.

ARR is recommended for the diagnosis of PA but is a highly variable test. In clinical practice, the factor most difficult to control but frequently encountered is that of a patient with hypertension undergoing screening for PA on medications that interfere with the measurement of the ARR. Robust detection of PA mandates that factors known to alter the ARR are controlled before sampling. Washout of interfering antihypertensive medications in non-hospitalized patients is not without risk; it is safe only in mildly hypertensive and regularly monitored patients. The key objective of the present study was to develop a diagnostic model for pre-washout screening of PA that is convenient for clinical application. Our analysis yielded the PAPS_3_ model, which has potential application in clinical practice and good predictive ability. However, PA should not be automatically diagnosed in patients who meet the PAPS_3_ scoring criteria; the recommended guidelines for PA screening, including an in-depth patient evaluation for possible PA, should still be followed. For patients able to undergo washout, the processes recommended by the guidelines for screening and confirmation should be followed. However, for patients who have difficulty tolerating washout of interfering drugs, the PAPS_3_ model can be recommended for the preliminary diagnosis of PA to reduce the risks to the patient during washout.

## Data Availability

The datasets generated during and/or analysed during the current study are available from the corresponding author on reasonable request.
